# Psychological correlates of physical activity among adults living in rural and urban settings

**DOI:** 10.3389/fpsyg.2024.1389078

**Published:** 2024-04-10

**Authors:** Zoe Sirotiak, Angelique G. Brellenthin, Arjun Hariharan, Amy S. Welch, Jacob D. Meyer, Warren D. Franke

**Affiliations:** ^1^Department of Kinesiology, Iowa State University, Ames, IA, United States; ^2^Department of Health and Human Performance, Norwich University, Northfield, VT, United States

**Keywords:** older adults, physical activity, psychological factors, rural health, urban health

## Abstract

Middle-aged and older adults living in rural settings have been consistently less likely to report regular physical activity (PA) than those living in urban settings. While past literature has identified sociodemographic and environmental correlates of PA that may contribute to these differences, consideration of psychological correlates has been limited. A total of 95 rural and urban adults ≥50 years old provided self-reported sociodemographic information, PA level, and psychological correlates of PA including measures assessing motivation, self-efficacy, social support, and attitudes related to PA. The average participant age was 68.6 years, and most were female (62.1%) and married (70.5%). While PA level did not differ significantly between the rural and urban groups, different psychological correlates contributed significantly to separate rural and urban linear regression models considering PA status. Among rural adults, more positive attitudes toward PA, and greater PA self-efficacy and social support were associated with greater amounts of PA while for urban adults, no psychological correlates were significantly associated with PA. Psychosocial factors may be key considerations in developing more effective PA interventions in middle-aged and older adults living in rural areas.

## Introduction

The benefits of regular physical activity (PA) are well documented in middle-aged and older adults (Langhammer et al., 2018; Centers for Disease Control and Prevention, 2023a). PA has an important role in helping adults preserve independence, control weight, prevent or manage chronic disease, and maintain muscle, joint and bone health (Sun et al., 2013; Langhammer et al., 2018; Eckstrom et al., 2020). PA has also been used in the prevention and management of many chronic health conditions affecting a substantial proportion of middle-aged and older adults, including cardiovascular disease, diabetes, and cancer (Anderson and Durstine, 2019). Although they can benefit from PA, middle-aged and older adults are the least physically active of any age group in the United States (Elgaddal et al., 2020), and there are further inequities in PA across many sociodemographic factors, including geographic location (Whitfield et al., 2023). Compared to urban or metropolitan older adults, fewer rural adults participate in regular PA (Cohen et al., 2018; Pelletier et al., 2021; Whitfield et al., 2023).

Several studies suggest that rural middle-aged and older adults are consistently less active than their urban counterparts (Parks et al., 2003; Martin et al., 2005; Cohen et al., 2018; Pelletier et al., 2021; Whitfield et al., 2023). However, other studies have noted higher levels of specific types of PA among adults living in more rural environments, such as recreational cycling (Arnadottir et al., 2009; Van Dyck et al., 2011; Fan et al., 2014). PA may be particularly important in rural communities, as rural adults are more likely to die from a variety of chronic diseases associated with physical inactivity including heart disease, cancer, and chronic lower respiratory disease (Centers for Disease Control and Prevention, 2023b). PA has been associated with health-related quality of life and mental health among adults living in rural areas (Hart, 2016; Smáradóttir et al., 2020). Limitations in the built environment and other structural barriers may be key drivers of rural–urban differences in PA (Brownson et al., 2009; Ferdinand et al., 2012; Sallis et al., 2020). However, improving the built environment in rural areas may not be sufficient to increase PA participation; other cultural, social, or psychological factors must be considered (Brownson et al., 2000; Trost et al., 2002; Wilcox et al., 2003; Koeneman et al., 2011; Perrin et al., 2016). Participating in regular PA is a complex behavior influenced by many factors (Trost et al., 2002; Park et al., 2015; Pelletier et al., 2021). In addition to the built environment, psychological determinants of PA, such as attitudes, expectations, beliefs, and knowledge of PA, are powerful predictors of long-term participation in PA among middle-aged and older adults (Harris et al., 2009; van Stralen et al., 2009; Kosteli et al., 2016).

While the built environment has been extensively considered in the context of PA differences between rural and urban adults, the associations of psychological factors in these contexts are unclear (Schutzer and Graves, 2004; Newson and Kemps, 2007; Kosteli et al., 2016). Psychological correlates of PA have been identified among older adults, including motivation for PA, social support for PA, and PA self-efficacy (Booth et al., 2000; Shores et al., 2009; Ayotte et al., 2010). However, comparisons of psychological correlates of PA between rural and urban adults may allow determination of specific psychological correlates that may be particularly beneficial in each setting. Identifying specific psychological correlates of PA in rural and urban older adults would allow targeted PA promotion programs for these individual populations. Thus, the purpose of this study is to assess psychological correlates of PA among rural and urban adults.

## Methods

### Participants

Participants were adults ≥50 years old who attended mobile health screening clinics at various locations across Iowa. Participants were invited to complete an optional PA questionnaire while waiting for their health screening. The survey probed PA participation and primary theoretical constructs underlying PA behavior (i.e., socio-ecological model, social cognitive theory, theory of planned behavior, and motives for PA). This study was approved by the Institutional Review Board and participants provided informed consent prior to data collection. Questionnaires were provided in the same order to each participant, and the questionnaires took approximately 10–15 min to administer. Participants were encouraged to contact the research team with any questions or concerns.

### Rural or urban designation

The U.S. Health Resources and Services Administration guidelines (U.S. Health Resources and Services Administration, 2022), which combine US Census, Office of Management and Budget, and Rural–Urban Commuting Area classifications, were used to identify rural areas as described in previous studies (Chrisman et al., 2014; Abildso et al., 2021). Participants living in zip codes identified to be rural were assigned a “rural” label while those outside of these zip codes were assigned an “urban” label.

### Primary outcome

#### Physical activity

The PA was measured using the Physical Activity Scale for the Elderly (PASE) (Washburn et al., 1993). The PASE is a brief instrument designed specifically to assess PA in older people over the past week. PASE is a reliable and valid measure of PA in older adults (α = 0.75) (Washburn et al., 1993, 1999).

Perceptions of an individual’s neighborhood were assessed with the Neighborhood Environment Walkability Scale (NEWS) (Saelens et al., 2003; Cerin et al., 2006). Three subscales of this measure were utilized including residential density, land use mix-access, and neighborhood satisfaction. Residential density was measured using six items inquiring about the types of buildings in an individual’s neighborhood rated on a 5-point Likert scale (“none” to “all”). Land use mix-access was assessed with seven items inquiring about access to various locations in their neighborhood (i.e., local stores, transit stops). These items are rated on a 4-point Likert scale ranging from “strongly disagree” to “strongly agree.” Finally, general neighborhood satisfaction was measured with a 17-item 5-point Likert scale. Items assessing perceptions of an individual’s neighborhood were rated from “strongly dissatisfied” to “strongly satisfied.” Internal consistencies were acceptable in the current sample (residential density α = 0.68; land use mix-access α = 0.73; neighborhood satisfaction α = 0.94).

### Primary predictors

We assessed numerous psychological variables that have previously been shown to be associated with PA. We included psychological questionnaires, described in more detail below, that assessed motivation, intention, self-efficacy, barriers, enablers, social support, perceptions, and attitudes toward PA. Given the theoretical similarities between the psychological scales, a principal components analysis (PCA) was used to identify distinct psychological correlates of PA and the resulting components used as the primary predictors are described below.

#### Component 1: motivation for PA

Motivation for PA was assessed using the Motives for Physical Activity Measure - Revised (MPAM-R) (Ryan et al., 1997). The five motives assessed were (1) fitness, or being physically active out of the desire to be physically healthy, strong, and energetic; (2) appearance, or being active to become more physically attractive, to have defined muscles, to look better, and to achieve or maintain a desired weight; (3) competence/challenge, or being physically active because of the desire to improve at an activity, to meet a challenge, and to acquire new skills; (4) social, or being physically active in order to be with friends and meet new people; and (5) enjoyment, or being physically active because it is fun, increases happiness, and is interesting, stimulating, and enjoyable (Ryan et al., 1997). Internal consistencies were acceptable in the current sample (fitness *α* = 0.95; appearance *α* = 0.91; competence *α* = 0.94; social *α* = 0.90; enjoyment *α* = 0.94).

Decisional balance, or the pros and cons of PA, was determined using a 16-item questionnaire, with 10 items pertaining to pros of PA while 6 items measured cons of PA (Marcus et al., 1992). Participants rated how each item affected their decision to engage in regular PA. The pros items were summed to produce raw scores that could range from 10 to 50, while the cons items were summed to produce raw scores that could range from 6 to 30. Internal consistency was adequate in our sample (pros *α* = 0.97; cons *α* = 0.76).

#### Component 2: PA self-efficacy

Intention to engage in PA and perceived behavioral control of PA, concepts of the Theory of Planned Behavior, were measured using questions from a previous study involving older adults (Gretebeck et al., 2007). Perceived behavioral control was measured with 3 items rating the ease or difficulty and amount of control they had over performing PA on a Likert scale. Internal consistency was adequate in our sample (*α* = 0.80). Intention was measured with 2 items in which participants rated their likelihood and intention of being physically active for 30 min, 3 days/week. Higher ratings reflected greater intention to participate in PA. Internal consistency was acceptable in our sample (*α* = 0.91).

Self-efficacy was assessed through two measures. The Exercise Self-Efficacy Scale (McAuley, 1993) is an 8-item measure that considers the participant’s beliefs in their ability to continue exercising at a moderate intensity for 3 times/week for 40 min or more over the next month. Internal consistency was acceptable in our sample (*α* = 0.96). Barrier self-efficacy for PA was assessed using a 12-item scale (McAuley and Mihalko, 1998) investigating adults’ perceived capabilities to exercise three times/week for the next 3 months in the face of barriers (e.g., bad weather, lack of interest/boredom, pain, and discomfort). Internal consistency was adequate in our sample (*α* = 0.97).

#### Component 3: social support for PA

Social support for PA was assessed using a 20-item questionnaire in which participants rated how often family (5 items) and friends (15 items) engaged in acts that were supportive of PA in the past 3 months, from 1 (never) to 5 (very often) (Sallis et al., 1987). The mean of the scores were calculated, with higher scores indicating greater social support. Internal consistency was adequate in the current sample (family *α* = 0.94; friends *α* = 0.93).

#### Component 4: attitudes toward PA

Instrumental attitude and affective attitude regarding PA, concepts of the Theory of Planned Behavior, were measured using questions from a previous study involving older adults (Gretebeck et al., 2007). The attitude toward PA scales were measured with 8 items using a 7-point semantic differential bipolar adjective scale (from −3 to 3). Positive scores indicated a more optimistic attitude toward PA. Internal consistency was acceptable in our sample (*α* = 0.94).

### Covariates

Participants self-reported sociodemographic and health characteristics including age, gender, education, marital status, health rating, height, and weight.

### Statistical analysis

Given the theoretical similarities between the psychological scales, a principal components analysis (PCA) was used to identify distinct psychological correlates of PA. Missing data were addressed at the individual measure level, with individual measure scores imputed from the average of completed rural and urban scales. The number of imputations varied by scale: PASE: 1 (1.1%), barriers self-efficacy: 3 (3.2%), PA self-efficacy: 4 (4.2%), instrumental attitude: 9 (9.5%), affective attitude: 10 (10.5%), perceived behavioral control: 2 (2.1%), intention: 2 (2.1%), pros: 4 (4.2%), social support- family: 7 (7.4%), social support- friends: 8 (8.4%), motives- interest: 4 (4.2%), motives- competence: 3 (3.2%), motives- appearance: 4 (4.2%), motives- fitness: 4 (4.2%), and motives-social: 4 (4.2%). Imputation was performed prior to PCA analysis. As our sample size precludes inclusion of numerous covariates, demographic and health-related variables were examined for inclusion as covariates in the linear regression models based on relationships with PA using bivariate correlations. Following PCA analysis, individual linear regression models assessing the associations of the PCA components with PA level (PASE) were performed for the combined group, as well as separate rural and urban subgroups.

## Results

### Preliminary analysis

A PCA was performed with the summary scores of 16 measures of hypothesized correlates of PA in 95 participants. The cons of PA subscale and subjective norm item were dropped from the analysis as neither had a correlation coefficient >0.3 with any other scale, leaving 14 measures in the final PCA. The data was assessed for suitability of PCA prior to analysis. The correlation matrix demonstrated that all remaining measures had at least one correlation coefficient greater than 0.3 and the overall Kaiser–Meyer–Olkin (KMO) value was 0.84. Bartlett’s test of sphericity was statistically significant (*p* < 0.001), indicating data factorizability. The PCA reported four components with eigenvalues greater than one which explained 45.91, 11.32, 10.00, and 9.33% of the total variances, respectively. Scree plot visualization suggested the retention of four components (Cattell, 2023). Therefore, four components were retained.

The four-component model explained 76.56% of the total variance. A Varimax with orthogonal rotation assisted with interpretation. The interpretation of the data was consistent with identified correlates of PA with strong loadings of motivation for PA on Component 1, PA self-efficacy on Component 2, social support for PA on Component 3, and attitudes toward PA on Component 4. PCA results can be found in Table 1.

**Table 1:** Principal components analysis results role of psychological correlates in explaining PA in combined, rural, and urban older adults.

	Factor 1	Factor 2	Factor 3	Factor 4
Motivation- fitness	**0.84**	0.31	−0.03	0.14
Motivation- appearance	**0.81**	0.25	0.11	0.09
Motivation- competence	**0.81**	0.22	0.32	0.13
Motivation- interest or enjoyment	**0.78**	0.17	0.34	0.25
Exercise pros	**0.74**	0.26	−0.08	0.05
Motivation- social	**0.62**	−0.16	0.51	0.28
Perceived behavioral control	0.16	**0.83**	0.05	0.23
Intention to exercise	0.19	**0.83**	0.04	0.20
PA self-efficacy	0.45	**0.76**	0.27	−0.07
Barriers self-efficacy	0.35	**0.62**	0.28	0.20
Social support- friends	0.09	0.06	**0.84**	0.07
Social support- family	0.12	0.30	**0.80**	0.04
Affective attitude	0.17	0.18	0.12	**0.88**
Instrumental attitude	0.17	0.21	0.04	**0.88**

Factor 1 represents motivation for PA, Factor 2 represents PA self-efficacy, Factor 3 represents social support for PA, and Factor 4 represents attitudes toward PA. Motivation- fitness, Motivation – appearance, Motivation- competence, Motivation- social, and Motivation- interest or enjoyment measured by Motives for Physical Activity Measure-Revised (MPAM-R). Exercise pros measured by decisional balance items described in Marcus et al. (1992). Perceived behavioral control, Intention to exercise, Instrumental attitude, and Affective attitude measured as described in Gretebeck et al. (2007). PA self-efficacy measured by Exercise Self-Efficacy Scale. Barriers self-efficacy measured as described by McAuley and Mihalko (1998). Social support- friends and social support- family measured as described by Sallis et al. (1987).

Bolded items indicate the PCA Factor with which each item loads on the most.

Bivariate correlations between PA and age, gender, education, marital status, overall health, and body mass index (by calculating weight (kg)/height (m^2^)) were each assessed, but only age was significantly correlated with PA [*r*(95) = −0.44, *p* < 0.001]. Therefore, only age was included as a covariate in the primary regression models.

### Primary analyses

Table 2 lists baseline characteristics of the rural and urban participants. In general, urban participants were more likely to be male with greater educational attainment than rural participants. Rural participants were more likely to be married, while urban participants were more likely to be widowed. There was no significant difference in PA levels between urban and rural adults [urban *M*(*SD*) = 175.12 (82.65); rural *M*(*SD*) = 168.70 (105.69); *t*(93) = −0.33; *p* = 0.742]. Middle aged adults (<65 years old) reported higher levels of PA compared to older individuals (≥65 years old) [*t*(94) = 4.09; *p* < 0.001]. Rural adults reported more positive attitudes toward PA than urban adults, as assessed by Component 4 of the PCA [*t*(92) = 3.90; *p* < 0.001] and affective attitude [*t*(93) = 3.14; *p* = 0.002] and instrumental attitude [*t*(93) = 2.17; *p* = 0.032] subscales. Urban adults reported significantly greater PA self-efficacy [*t*(93) = −2.22; *p* = 0.029]. All other individual scale comparisons were not significant between rural and urban groups.

**Table 2:** Descriptive characteristics of the sample.

	Total (*N* = 95)	Urban (*N* = 48)	Rural (*N* = 47)
Age, *M* years (*SD*)	68.55 (11.2)	67.63 (11.5)	69.49 (11.0)
Gender, *N* (%)			
Female	59 (62.1)	26 (54.2)	33 (70.2)
Male	36 (37.9)	22 (45.8)	14 (29.8)
Education^*^			
High school or less	40 (42.5)	16 (33.4)	24 (52.2)
Trade school	12 (12.8)	3 (6.3)	9 (19.6)
Some college	10 (10.6)	8 (16.7)	2 (4.3)
College degree or higher	32 (34.1)	21 (43.9)	11 (23.8)
Marital status, *N* (%)			
Never married	2 (2.1)	1 (2.1)	1 (2.1)
Married	67 (70.5)	36 (58.3)	31 (66.0)
Divorced or separated	10 (10.5)	5 (10.4)	5 (10.6)
Widowed	16 (16.8)	6 (12.5)	10 (21.3)
Overall health, *N* (%)			
Excellent	15 (15.8)	11 (22.9)	4 (8.5)
Good	62 (65.3)	28 (58.3)	34 (72.3)
Fair	16 (16.8)	8 (16.7)	8 (17.0)
Poor	2 (2.1)	1 (2.1)	1 (2.1)
BMI, *M* (*SD*)	27.95 (4.9)	27.93 (5.1)	27.98 (4.6)
PASE, *M* (*SD*)	171.94 (94.3)	175.12 (82.6)	168.70 (105.7)
Motivation- fitness, *M* (*SD*)	26.87 (8.1)	27.38 (7.5)	26.35 (8.7)
Motivation- competence, *M* (*SD*)	28.67 (11.6)	29.39 (11.7)	27.93 (11.6)
Motivation- interest or enjoyment, *M* (*SD*)	28.99 (12.0)	28.88 (11.9)	29.09 (12.3)
Motivation- appearance, *M* (*SD*)	27.47 (9.6)	28.30 (8.8)	26.62 (10.4)
Exercise pros, *M* (*SD*)	36.95 (10.5)	37.89 (9.5)	35.99 (11.6)
Motivation- social, *M* (*SD*)	16.19 (8.3)	15.19 (7.7)	17.21 (8.9)
Perceived behavioral control, *M* (*SD*)	3.55 (1.1)	3.62 (1.1)	3.47 (1.2)
Intention to exercise, *M* (*SD*)	3.47 (1.4)	3.53 (1.3)	3.41 (1.5)
PA self-efficacy, *M* (*SD*)^*^	41.47 (33.0)	48.75 (33.4)	34.04 (31.3)
Barriers self-efficacy, *M* (*SD*)	38.09 (26.7)	37.85 (26.7)	38.34 (27.00)
Social support- friends, *M* (*SD*)	1.69 (0.9)	1.73 (0.9)	1.65 (0.85)
Social support- family, *M* (*SD*)	2.10 (1.0)	2.09 (1.0)	2.11 (1.1)
Instrumental attitude, *M* (*SD*)^*^	2.13 (1.2)	1.87 (1.36)	2.39 (0.9)
Affective attitude, *M* (*SD*)^*^	1.68 (1.4)	1.26 (1.5)	2.10 (1.0)

^*^Indicates significant difference (*p* < 0.05) between groups. BMI, body mass index. PASE, Physical Activity Scale for the Elderly. Motivation- fitness, Motivation – appearance, motivation- competence, motivation- social, and motivation- interest or enjoyment measured by Motives for Physical Activity Measure-Revised (MPAM-R). Exercise pros measured by decisional balance items described in Marcus et al. (1992). Perceived behavioral control, Intention to exercise, Instrumental attitude, and Affective attitude measured as described in Gretebeck et al. (2007). PA self-efficacy measured by Exercise Self-Efficacy Scale. Barriers self-efficacy measured as described by McAuley and Mihalko (1998). Social support- friends and social support- family measured as described by Sallis et al. (1987).

Results of the combined final model indicated that age [*β* = −0.39, *t*(93) = −4.24, *p* < 0.001] and PA self-efficacy [*β* = 0.29, *t*(93) = 3.36, *p* = 0.001] significantly contributed to PA level (Table 3). For the rural sample, age [*β* = −0.31, *t*(46) = −2.54, *p* = 0.015], self-efficacy [*β* = 0.28 *t*(46) = 2.41, *p* = 0.020], social support [*β* = 0.34, *t*(46) = 2.78, *p* = 0.008], and attitudes toward PA [*β* = 0.29, *t*(46) = 2.50, *p* = 0.017] were each significantly associated with PA level. In the urban sample, only age [*β* = −0.51, *t*(47) = −3.63, *p* < 0.001] was significantly associated with PA level. Detailed regression results can be found in Table 3.

**Table 3:** Role of psychological correlates in explaining PA in combined, rural, and urban older adults.

Variable	*B*	*SE*	*β*	*t*	*p*	
Combined						*R*^2^ = 0.37^**^
**Age**	**−3.34**	**0.79**	**−0.39**	**−4.24**	**<0.001**	
Motivation for PA	8.95	8.09	0.10	1.11	0.272	
**Self-efficacy**	**27.45**	**8.16**	**0.29**	**3.36**	**0.001**	
Social support	15.56	8.43	0.17	1.85	0.068	
Attitudes toward PA	15.54	8.01	0.17	1.95	0.056	
Rural						*R*^2^ = 0.49^**^
**Age**	**−3.01**	**1.18**	**−0.31**	**−2.54**	**0.015**	
Motivation for PA	1.48	11.38	0.02	0.13	0.897	
**Self-efficacy**	**28.66**	**11.89**	**0.28**	**2.41**	**0.020**	
**Social support**	**34.63**	**12.47**	**0.34**	**2.78**	**0.008**	
**Attitudes toward PA**	**44.30**	**17.75**	**0.29**	**2.50**	**0.017**	
Urban						*R*^2^ = 0.36^**^
**Age**	**−3.83**	**1.06**	**−0.51**	**−3.63**	**<0.001**	
Motivation for PA	17.06	11.47	0.20	1.49	0.144	
Self-efficacy	20.86	11.12	0.24	11,88	0.068	
Social support	−4.20	10.84	−0.05	−0.39	0.701	
Attitudes toward PA	5.21	9.16	0.07	0.57	0.572	

^**^p < 0.001. Physical activity as assessed through PASE is the outcome measure for each model. Motivation for PA representing Factor 1, self-efficacy representing Factor 2, social support representing Factor 3, and attitudes toward PA representing Factor 4. Bolded items contribute significantly to the model.

### Sensitivity analyses

Gender and education were explored as covariates in conjunction with age in the regression models given their established association with physical activity (Plotnikoff et al., 2004). With these added covariates, the attitudes toward PA component was also significantly associated with PA (*p* = 0.021) in the combined model. There were no significantly different conclusions in the rural or urban stratified models.

In an additional sensitivity analysis, we assessed associations of available neighborhood environment-related subscales with PA. Neighborhood Environment Walkability Scale (Saelens et al., 2003; Cerin et al., 2006) subscales were available for a subset of participants in our sample (residential density *n* = 78; land use mix-access *n* = 60; neighborhood satisfaction *n* = 89). Neither residential density, described as the perceived density of residences in an area, or land use mix-access, defined as the perceived ability to access places nearby through PA, differed between rural and urban groups [*t*(76) = 0.06, *p* = 0.949; *t*(58) = −0.60, *p* = 0.552]. However, rural adults reported greater neighborhood satisfaction than urban adults [*t*(87) = 2.45, *p* = 0.016]. Therefore, neighborhood satisfaction was included in a regression model with age and the PCA components. In the combined model, social support was significantly associated with PA in addition to age and self-efficacy. Social support (*p* = 0.012) and attitudes toward PA (*p* = 0.028) remained significant in the rural model. There were no significant predictors in the urban subsample (*p*s > 0.05).

## Discussion

### PA among rural and urban adults

This study aimed to identify psychological correlates of PA in rural and urban middle aged and older adults. Middle aged adults reported higher levels of PA compared older adults, fitting with prior literature (Spartano et al., 2019). We found similar levels of PA in rural and urban participants, which contrasts with other studies reporting that rural residents engage in less PA than their urban counterparts (Cohen et al., 2018; Pelletier et al., 2021; Whitfield et al., 2023). Possible reasons for similar PA levels in our rural and urban participants include similar perceived residential density and land use mix-access, as well as greater neighborhood satisfaction among rural participants although this was not associated with PA in any final regression models. Lack of perceived differences in location accessibility and residential density, as well as greater neighborhood satisfaction may have caused our rural subset to be more active than rural groups in previous literature (Stronegger et al., 2010; Wang et al., 2019). Age was also the strongest predictor of PA levels, and age was similar between our urban and rural samples, which has not always been the case in previous studies (Parks et al., 2003; Martin et al., 2005). Finally, while we followed HRSA rural–urban definitions, the range of populations for the rural and urban locales may not have been as extreme as in other studies. For example, the ‘urban’ zip codes in this study had an average population of 211,754 people while the rural areas averaged 25,934 people (US Census Bureau, 2010; U.S. Health Resources and Services Administration, 2022). Previous work reporting on rural–urban differences in PA has included much larger metro areas of 1–5+ million residents (US Census Bureau, 2010; Cohen et al., 2018). Thus, it is quite possible that Iowa is relatively more homogenous in its population density, which could contribute to less separation in rural and urban characteristics.

### Psychological correlates of PA

Despite finding no differences in self-reported PA levels, different psychological correlates of PA were identified in stratified analyses performed with rural and urban participants. Among rural participants, greater social support, and self-efficacy, as well as more positive attitudes toward PA were associated with greater amounts of PA. This finding aligns with reports of social support barriers associated with lower PA levels among rural adults, while not urban adults (Pelletier et al., 2022). Among urban participants, however, none of the psychological correlates were significantly associated with PA. These results suggest that the association between psychological correlates and PA may differ based on rural or urban residence. Resources targeting psychological correlates of PA may be particularly impactful among rural adults, while PA may be more affected by other factors in urban adults such as the individual’s physical health and environment (Weiss et al., 2007; Sallis et al., 2012).

Interestingly, motivation for exercise, while the leading component identified by the PCA analysis accounting for nearly half of the total variance, was not significantly associated with PA in the combined, rural, or urban models conflicting with findings in prior rural and urban samples (Pelletier et al., 2022). This finding suggests that motivation, which has been viewed as a vital predictor of PA performance and public health promotion target (Teixeira et al., 2012; Molanorouzi et al., 2015), may not be the primary driver of PA behavior in middle-aged and older adults. However, our results do fit with other literature demonstrating that motivation is a weaker predictor of PA than other psychological correlates, perhaps because motivation can be transient (Ryan and Deci, 2000; Braver et al., 2014). Social cohesion has been noted among many rural communities (Avery et al., 2021), and considering group attitudes and social support may be particularly important among individuals living in rural areas. On the other hand, urban areas may have more built-in support for PA with extensive infrastructure for PA, making social support and group attitudes less important in predicting PA level among urban adults. Our results indicate that considering the environment and characteristics of the targeted population and targeting self-efficacy, social support, and attitudes toward PA may produce more effective health promotion campaigns. While many PA promotion programs have targeted the built environment and motivation for PA (Brownson et al., 2009; Ferdinand et al., 2012; Sallis et al., 2020), our findings indicate that interventions targeting self-efficacy, social support, and attitudes toward PA may be more impactful, particularly among rural adults. Further research is needed to develop these intervention programs.

### Limitations

There were limitations to the present study. The study was cross-sectional, and it is possible that higher levels of PA may exert reciprocal influences on positive psychosocial correlates of PA, distinct from the impact of rural or urban setting. The sample size was limited, and our findings cannot be extrapolated to all rural and urban middle-aged and older adults. PA was self-reported, which is prone to reporting bias.

## Conclusion

In summary, rural middle-aged and older adults have been consistently noted to perform less PA than their urban peers and while sociodemographic and environmental correlates have been considered in prior work, there has been little investigation of potential psychological considerations. Our findings suggest that self-efficacy, social support, and positive attitudes toward PA are significantly associated with PA in rural populations, while psychological correlates were not significantly associated with PA in urban populations. Considering these findings in identifying those at risk of low PA levels and while developing more effective and specific PA campaigns may assist in improving health in these populations. Future studies could investigate interventions targeting distinct, specific psychological correlates in rural and urban areas to assist middle-aged and older adults in increasing PA levels and health. Intervention development in improving self-efficacy, social support, and attitudes toward PA among rural adults may be particularly impactful future research. Receiving community input through focus group discussions and an iterative study design may allow identification of community factors to consider in intervention development and implementation. Evaluating community factors will allow identification of important considerations prior to implementation of costly built environment or other intensive intervention. Our results indicate that considering psychological variables rather than solely the built environment may provide impactful PA interventions, particularly among rural adults.

## Data availability statement

The raw data supporting the conclusions of this article will be made available by the authors, without undue reservation.

## Ethics statement

This study involving humans was approved by the Iowa State University Institutional Review Board. The study was conducted in accordance with local legislation and institutional requirements. The participants provided their written informed consent to participate in this study.

## Author contributions

ZS: Formal analysis, Methodology, Writing – original draft. AB: Formal analysis, Methodology, Supervision, Writing – review & editing. AH: Conceptualization, Investigation, Methodology, Writing – original draft. AW: Data curation, Investigation, Supervision, Writing – review & editing. JM: Methodology, Writing – review & editing. WF: Conceptualization, Data curation, Methodology, Supervision, Writing – review & editing.
